# Brain Tumor Analysis Empowered with Deep Learning: A Review, Taxonomy, and Future Challenges

**DOI:** 10.3390/brainsci10020118

**Published:** 2020-02-22

**Authors:** Muhammad Waqas Nadeem, Mohammed A. Al Ghamdi, Muzammil Hussain, Muhammad Adnan Khan, Khalid Masood Khan, Sultan H. Almotiri, Suhail Ashfaq Butt

**Affiliations:** 1 Department of Computer Science, Lahore Garrison University, Lahore 54000, Pakistan; madnankhan@lgu.edu.pk (M.A.K.); khalid.masood@lgu.edu.pk (K.M.K.); 2 Department of Computer Science, School of Systems and Technology, University of Management and Technology, Lahore 54000, Pakistan; muzammil.hussain@umt.edu.pk; 3 Department of Computer Science, Umm Al-Qura University, Makkah 23500, Saudi Arabia; maeghamdi@uqu.edu.sa (M.A.A.G.); shmotiri@uqu.edu.sa (S.H.A.); 4 Department of Information Sciences, Division of Science and Technology, University of Education Township, Lahore 54700, Pakistan; suhail.ashfaq@ue.edu.pk

**Keywords:** deep learning, brain tumor, computer vision, bioinformatics, segmentation, medical images, review

## Abstract

Deep Learning (DL) algorithms enabled computational models consist of multiple processing layers that represent data with multiple levels of abstraction. In recent years, usage of deep learning is rapidly proliferating in almost every domain, especially in medical image processing, medical image analysis, and bioinformatics. Consequently, deep learning has dramatically changed and improved the means of recognition, prediction, and diagnosis effectively in numerous areas of healthcare such as pathology, brain tumor, lung cancer, abdomen, cardiac, and retina. Considering the wide range of applications of deep learning, the objective of this article is to review major deep learning concepts pertinent to brain tumor analysis (e.g., segmentation, classification, prediction, evaluation.). A review conducted by summarizing a large number of scientific contributions to the field (i.e., deep learning in brain tumor analysis) is presented in this study. A coherent taxonomy of research landscape from the literature has also been mapped, and the major aspects of this emerging field have been discussed and analyzed. A critical discussion section to show the limitations of deep learning techniques has been included at the end to elaborate open research challenges and directions for future work in this emergent area.

## 1. Introduction

The advancement in medical technologies helps the clinical experts to facilitate more efficient e-health care systems to the patients. There is a number of medical domains where e-health care systems are beneficial [1]. Computer vision-based applications of biomedical imaging are gaining more importance as they provide recognition information to the radiologist for batter treatment-related problems. Different medical imaging techniques and methods that include X-ray, Magnetic Resonance Imaging (MRIs), Ultrasound, and Computed Tomography (CT), have a great influence on the diagnosis and treatment process of patients [2,3].

The formation of abnormal groups of cells inside the brain or near it leads to the initialization of a brain tumor. The abnormal cells abrupt the processing of the brain and affect the health of a patient [4]. Brain imaging analysis, diagnosis, and treatment with adopted medical imaging techniques are the main focus of research for the researcher, radiologist and clinical experts [5]. The analysis of brain images is considered imperative because diseases of the brain called brain tumors are fatal and responsible for a large number of deaths in developed countries; for instance, according to the National Brain Tumor Foundation (NBTF), 29,000 people are diagnosed with brain tumor in the United States (US) with brain tumor and 13,000 of those patients die per annum [6]. A number of advanced Magnetic Resonance Imaging (MRI) techniques that include Diffusion Tensor Imaging (DTI), MR Spectroscopy (MRS) and Perfusion MR are used for the analysis of brain tumor through MRI [7,8,9]. Brain tumor is broadly classified into two types: cancerous tumors, known as malignant tumors, and noncancerous tumors, known as benign tumors. Malignant tumors are further classified into grades I to IV by World Health Organization (WHO) [10]. A Grade-I tumor is called Pilocytic Astrocytoma, Grade-II tumor is Low-Grade Astrocytoma, Grade-III tumor is Anaplastic Astrocytoma and Grade-IV tumor is Glioblastoma. Grade-I tumors and Grade-II tumors are semi-malignant tumors with less aggressiveness. Grade-III and Grade-IV are malignant tumors and highly affect the health of the patient and may lead to the death of tumor patients [11].

A variety of image-processing techniques and methods have been used for the diagnosis and treatment of a brain tumor. Segmentation is the fundamental step in image processing techniques and is used to extract the infected region of brain tissue from MRIs [12]. Segmentation of the tumor region is an important task for cancer diagnosis, treatment, and the evaluation of treatment outcomes. A vast number of semi-automatic and automatic segmentation methods and techniques are used for tumor segmentation [13]. MRI contains methods with multiple sequence that include T1-weighted (TI) and T1-weighted contrast-enhanced (T1c), T2-weighted and T2-weighted Fluid Attenuated Inversion Recovery (FLAIR) techniques, which are employed for the segmentation of brain tumor.

MRIs have various features that are adopted in brain tumor segmentation studies that include image textures [14], local histograms [15], and structure tensor eigenvalues [16]. Machine learning methods such as Support Vector Machines (SVMs) [17,18,19] and Random Forest (RF) [14,15,16,20] are commonly used for pattern classification in tumor segmentation studies. Deep-learning-based techniques and methods are becoming popular in brain tumor segmentation studies, as their performance is superior in image analysis fields, such as object detection [21], image classification [22] and semantic segmentation [23,24,25]. Deep learning techniques have achieved state-of-the-art performance for automatic segmentation of brain tumors through multi-model MRIs [1]. The Convolutional Neural Network (CNN) is a powerful method for image recognition and prediction. However, CNN is mostly used for brain tumor segmentation, classification, and prediction of survival time for patients [26,27,28]. More deep-learning-based methods that are utilized for tumor segmentation, classification, and prediction include Stacked De-Noising Autoencoders [29] and Convolutional Restricted Boltzman Machine [30]. Among all the deep learning methods and techniques, CNNs perform batter for image segmentation, classification, and prediction. Two-Dimensional CNNs (2D-CNNs) [31,32,33,34,35] and 3D-CNNs [16,36,37], were both adopted to build brain tumor segmentation, classification, and prediction methods. Segmentation methods classify the image patch into different classes, such as necrosis, healthy tissues, edema, enhancing core and non-enhancing core.

Different tumor cells show distinct phenotypic and morphological information for segmentation, classification, and prediction, including gene expression, motility, cellular morphology, metabolism metastatic potential, and proliferation. This paper presents a review of various methods, techniques, frameworks, architectures, algorithms and critical studies using deep learning for segmentation, classification, and survival time prediction. Survey taxonomy describes the methods, techniques, systems, algorithms, frameworks, and architectures that are based on tumor segmentation, evaluation, and features exploration for tumor prediction and its classification. The review performs an analysis of the features extraction techniques, dataset utilized, tools, languages, and libraries that are used for implementation, recognition and evaluation measures. The issues and research gaps in various existing research problems include the key issues in tumor recognition for monitoring, recognition procedures and treatment plans for cancer patients.

The application of deep learning to brain tumor analysis first appears in conferences and workshops, and then in journals. The number of research papers grew rapidly from 2015 to onward. This topic has now became dominant at different conferences and journals.

**Figure 1:**
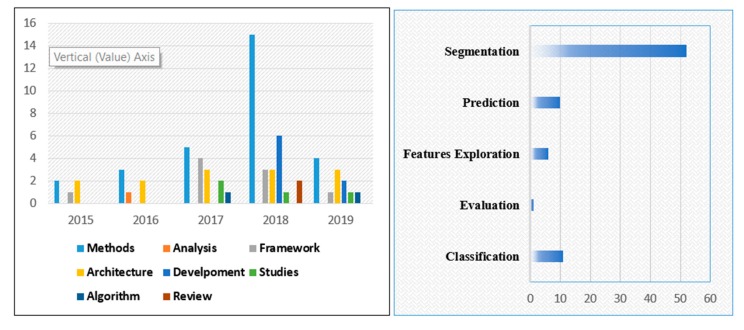
Breakdown of the papers included in this review in the year of publication.

**Figure 2:**
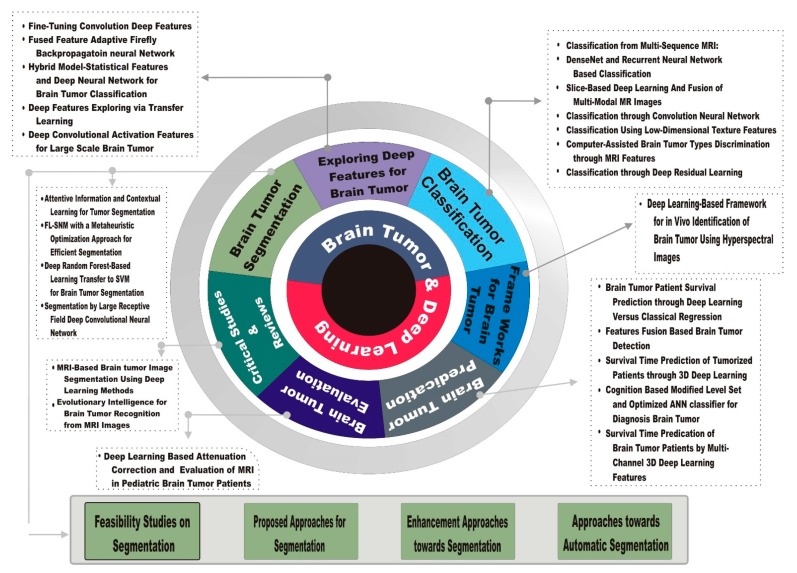
Literature Taxonomy of brain tumor using deep learning.

Figure 1 illustrates the development of deep learning applications to brain tumor analysis. Figure 2 presents a literature-based taxonomy for brain tumor analysis.

The development of deep learning application to brain tumor analysis motivated us to present a comprehensive review in all fields of brain tumor that includes segmentation, prediction, classification, both from a methodology-driven and applications perspective. The review also includes an overview of all the research publications in tabular form that helps readers to quickly assess the field. Consequently, this review presents a dedicated discussion section to the readers that covers the state-of-the-art successful development, open research challenges and an overview of future research directions. 

The review includes a large number of research papers, most of them recent, presenting an extensive variety of deep learning applications in brain tumor analysis to identify the most relevant contribution (“deep learning” AND “Brain Tumor”) in the title and abstract query performed. Additionally, MICCAI workshop papers related to brain tumors have also been included in this review. In summary, the aim of this review is (a) to show the deep learning development in the entire field of brain tumor, (b) the identification of open research challenges for successful deep learning methods for brain tumor tasks, (c) to highlight the successful deep learning contribution to brain tumor analysis.

## 2. Healthcare Scalability Importance and Challenges

The scalability in healthcare services, that includes the patient prioritization process and patient analysis, is a challenging task [38]. The demand for health care services is increasing gradually as the number of patients increases due to a rise in the population. The priority of healthcare services is based on the emergency status of patients. The identification of innovative research contributions for the provision of effective and efficient health care systems is an important and challenging task [39,40,41,42,43,44,45,46,47,48]. Various studies are conducted in bioinformatics to improve the prioritization process and provide a solution for the scalability problems in health care services [38,49].

This section introduces the relevant literature that explores the dilemma of the growing number of elderly patients who need timely and effective telemedicine services. An increase in the number of patients is expected to occur in the context of an ageing population [38,50,51,52,53,54,55] and disasters phenomena [56]. There are a number of problems in health care services but the aging population is considered to be the greatest problem [54,55,57,58].

**Figure 3:**
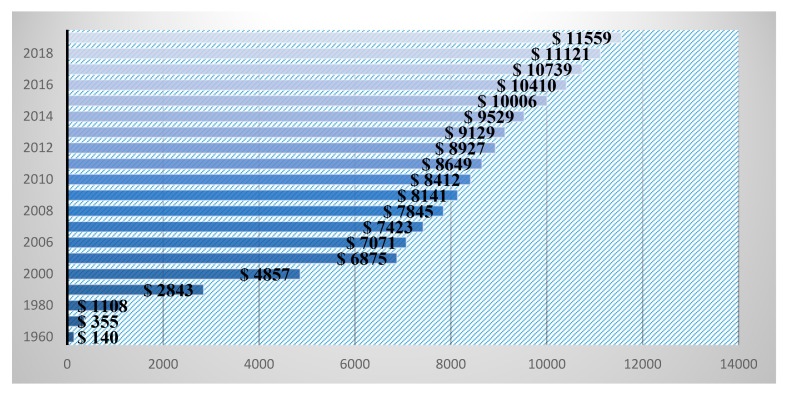
National healthcare expenditure per capita in the US.

The major changes in demographics lead to serious issues in the health care system [59]. As an increment in serious problems and permanent issues in the health care domain rises, the social and economic burdens increase [59,60,61]. Globally, health care systems and society loaded with burdens may result in a population’s aging problems. By 2030, 13% of the world population will fall in the aging category and the burden on the health care sector will be enormous [62]. Serious diseases that include brain tumors, chest cancer, lung cancer, diabetes, hypertension, and heart failure, directly affect medical health care expenses all over the world [63,64,65]. The manual treatment of serious disease is a challenging task for the global health care systems in terms of quality of care delivery [65,66]. As the number of patients in the health care domain increases, an increase in the United State (US) health care services expenditure is reported. The Center for Medicare and Medicaid Services (CMS) revealed that US health care expenditures gradually increases every year, as shown in Figure 3.

## 3. Brain Tumor Classification

The deep learning techniques and methods have performed well on image classification and supervised machine learning, as reported in recent research papers. Brain tumor has various classes, which include glioma, meningioma and pituitary tumors. Brain tumor is further classified as benign or low-grade I and II and malignant tumor, or high-grade III and IV. The following paragraphs thoroughly explain the recent research into brain tumor analysis. Table 1 shows the various data sources and their acquisition methods.

The classification of brain tumors is a challenging task due to variations in the shape, size, location, and contrast of tumor tissue cells. State-of-the-art deep learning techniques are used to classify different types of brain tumors—glioma, meningioma and pituitary types—that are further separated into axial, coronal and sagittal planes. Segmentation algorithms are employed for the extraction of features from axial slices using Dense CNN and these sequential features of multiple frames are classified by the recurrent neural network [68]. Generally, fully connected and convolutional networks are used in the classification models of brain tumors. The dataset, which is publicly available, contains 3064 enhanced contract brain MRIs and 989 axial images to minimize the error rate of neural networks in identifying the tumor. The test is performed on 512 × 512 axial images. Training is performed on axial images using five-fold cross-validation tests that increase the accuracy of the classification [75]. Table 2 describes the literature overview related to brain tumor classification.

The term cytotechnologist is used for experts who diagnose brain tumors. Astrocytes are a glia type cell of nerves and it is very difficult to differentiate between astrocyte and low-grade astrocytoma. The BING method is used to segment the cell regions and, for classification, convolution neural networks with residual learning are employed [73]. After detecting brain cells, the Voronoi diagram, watershed transform, and binarization are used in segmentation. Finally, CNN is performed on the segmented cells that achieve 98.5% classification accuracy [73]. The Extreme Learning Machine Local Receptive Fields (ELM-LRF) method is also proposed for the classification of tumors, which consists of three phases: removal of the noise using local and nonlocal methods, segmentation of benign or malignant tumor using ELM-LRF, and its classification. The cranial MR images are used in the proposed solution as they contain mass. The proposed method is effective and, using cranial MR images, an accuracy of 97.18% is achieved [71].

Misdiagnosis of the tumor affects the medical intervention and reduces the chances of survival of patients. Conventional methods that identify the tumor using MRIs are time-consuming for large datasets. The CNN architecture contains one layer each for max-pooling, flattening and convolutions, and these layers are fully connected with hidden layers that do not need any prior region-based segmentation. The architecture is trained on a publicly available dataset containing 3064 MRIs that achieve 98.51% accuracy for classification [69]. Three ConvNets-based models are proposed, in which a Convolutional Neural Network is trained through scratch, slices, patches and multiplanar volumetric slices of MRIs. Two ConvNets VGGNet and ResNet are trained by Images Net dataset and fine-tuning is used to train the last few layers of the ConvNet. The performance of the proposed ConvNet is tested using the Leave-One-Patient-Out (LOPO) scheme. ConvNet attains better accuracy compared to the existing models as it contains a self-learning feature with kernels/filters on different layers of ConvNet [67]. Oval multi-stream deep CNN architecture is proposed for brain tumor classification, in which molecular-related subcategories are utilized for tumor grades. Different enhanced and sensitive MRIs T1-MRI, T2-MRI, and FLAIR are used for fusion of the features in glioma grading classification. The objectives are achieved by the proposed architecture that employs multi-stream 2D deep CNN in glioma grading classification.

Fusion features are aggregated for scans of T1.MRI, T2.MRI and FLAIR brain images and 2D slices of 2D images are used to mitigate the over-fitting problems. The proposed architecture performs decently for grade glioma classification with 90.87% accuracy [135]. 

DNA methylation-based approaches that contain multi-modal medical images are used in the classification of glioblastomas tumors. 3D implementation, such as Histograms of Oriented Gradient (HOG), Local Binary Pattern (LBP) and Binary Robust Independent Elementary Features (BRIEF), is developed for short local image descriptors where tumor regions are identified by Bag-of-patterns as well as hand-crafted and auto-encoders deep features that are computed for segmentation masks in tumor diagnosis [70].

## 4. Brain Tumor Prediction

Prediction of brain tumors and the chances of survival for patients are open challenges for the researchers. MRIs opens ways of research in the field of brain tumors such as prediction, classification and segmentation analysis. Brain tumors are classified into two categories that consist of benign and malignant lesions. The multi-class tumors are also further subcategorized into XX and YY described from major to minor [72]. The size of the dataset is strongly linked with regression and other deep learning methods. The 3D-convolutional neural network plays an important role in classical regression methods for survival time prediction of patients with high-grade brain tumors. 3D CNN is used with Support Vector Classifier for better accuracy. Tumor cell shape, location, intensity and deep features are investigated during the experiment. More training data are required for the regression-based methods [83]. The survival time is varied in short-term, mid-term and long-term for high-grade gliomas tumor patients. A research study is carried out for the accuracy of different machine learning and deep leaning Brats 2017 dataset samples that consist of 163 samples of brain MRIs. Deep features that include intensity and statistical texture, and volumetric and shape of tumor cell are important for the training of various Machine Learning (ML) and Deep Learning (DL) methods. Different ML and DL methods that include Support Vector Machine (SVM), e, linear discriminant analysis, logistic regression and K-Nearest Neighbors (KNN) are tested on Brat’s dataset, and accuracies are compared. The best prediction accuracy is achieved using a hybrid algorithm combining CNN and linear discriminant analysis [87]. CNN is a well-known method for image recognition and prediction. MvNet and SPNet are used to address the challenges of multimodal tumor segmentation. Multi-view Network slices the multimodal images from different view-points, which consist of three Multi-branch layers that are fully connected with Residual Networks (Mb-FCRN). Mb-FCRN produces independent segmentation results and then SPNet is employed to measure the survival time for the temporized patients [84]. Table 3 shows an overview of the literature reports based on brain tumor prediction techniques using deep learning.

A two-stage learning-based method is proposed by D. Nie for the prediction of overall survival time for high-grade gliomas tumor patients. In the first stage, high-grade features are extracted to enhance multi-modal, multi-channel MRIs to increase the predicted survival time. Two-stage learning methods are used for contrast-enhanced MRIs as well as in Diffusion Tensor Imaging (DTI), and resting-state MRI images for computing different metric maps that include DTI images for generating diffusivity maps and anisotropy-related fluctuation frequency maps. The 3D convolutional neural network consists of multi-channel metric maps that are used to extract the high-grade predictive features from the individual patch of these maps, and trains the network layers for prediction. In the second stage, Support Vector Machine (SVM) are used to classify tumor-related features such as age, histological type, and tumor size to predict the final (short or long) overall survival time of high-grade gliomas patients with 90.66% accuracy [136].

The Extreme Learning Machine Local Receptive Fields (ELM-LRF) method is proposed for the prediction of tumors, containing three phases that include the removal of the noises from images by local and nonlocal methods, the prediction of benign or malignant tumor using ELM-LRF and segmentation of tumor. The cranial MR images are used in the proposed method, as the images have more mass. The proposed method is effective and gives a high accuracy of 97.18% for malignant tumors when cranial MR images use [71].

High-grade gliomas brain tumor is very aggressive and leads to the death of a patient in 1–2 years. The accurate and timely prognosis of the gliomas brain tumor increases chance of survival. The extraction of the deep features of gliomas patients from MRI, DTI, and fMRI is important for prediction of overall survival time. 3D CNN with multi-channel data extracts the deep and clinical features, and using SVM predicts short, long and overall survival times of the gliomas patients [85]. 

The variable and complex shapes, textures, sizes, and locations of brain tumor cells are a few challenges for automatic detection of the tumor. An unsupervised clustering method that has a fused feature vector is a mixture of the Local Binary Pattern (LBP), Gabor Wavelet Features (GWF), Histograms of Oriented Gradient (HOG) and Segmentation-Based Fractal Texture Analysis (SFTA) are developed by J. Amin for the prediction of brain tumor. Random Forest (RF) is used with 0.5 holdout cross-validation to avoid overfitting problem in the prediction and classification of tumors into complete, enhancing and non-enhancing regions [86].

Neuro endoscopy and invasive procedures have great impact on the prediction and treatment of pituitary brain tumors. The Eyebrow Orbitotomy approach is used by neurosurgery and assistant surgeons to predict the brain tumor [137]. 

Another approach is presented for the classification of brain tumor in which a modified level set method is used to segment the tumor region. The feature set thr Gabor and moment invariant, and Grey Level Co-Occurrence Matrix (GLCM), that are extracted using Multi-Level wavelet decomposition. After features selection, Adaptive Artificial Neural Network (AANN) is applied on selected features for the prediction of brain tumor. To increase the accuracy of the ANN, optimization for layers of the network is performed using the Whale Optimization Algorithm (WOA) [88].

## 5. Exploring Deep Features for Brain Tumor

Deep features exploration and representation is an important task for the prediction and diagnosis of brain tumor from radiological MRIs. Deep features are extracted from MRI images for diagnosis, therapy, and prognosis in oncology. The radiomic properties of the images clearly link with meaningful biological characteristics and give qualitative pieces of information that are familiar to radiologists [138]. Deep convolutional neural networks achieve state-of-the-art performance for prediction and classification when network is pre-trained as features extractor. Deep feature extractor methods and techniques are better for the prediction of over-all survival time for the tumorized patients [80]. Deep Convolutional Neural Networks (CNNs) activation method is used to extract the features from ImageNet to train the CNNs networks for classification and segmentation. CNN’s activation features method employs various techniques that include features’ selection, features’ pooling, and data augmentation algorithms [76].

To reduce the intensity of variation of the images’ different average filters, features selection, features extraction and fusion are performed. Gabor Wavelet features technique is used to obtain the texture information of the image that contains the locality orientation and frequency of the tumor. Kernel Principal Component Analysis (KPCA) selects the small subset of the features and reduces the redundancy by increasing the relevancy of the features. Gaussian Radial Basis Function (GRBF) gives distinguished information of features from multiple sets of features for feature fusion [78]. Fine-tuning-based feature extraction is used in the pre-trained CNNs method. Fine-tuned CNNs are initially trained with a large amount of natural image data and then adopt features representation that is used for different brain tumor containing segmentation, classification, and prediction of survival time for tumorized patients [77]. Table 4 shows the overview of the literature.

## 6. Brain Tumor Segmentation

Brain tumor segmentation is performed to extract the tumor region from the images for the further classification and prediction of brain tumors. Different Machine ML/DL methods are proposed for the segmentation of tumorized cells. Some of these ML methods use manually segmented images for the training, which is costly, time-consuming and needs medical expertise. Two types of data, fully-annotated and weakly annotated data, train the deep learning methods for segmentation. A method that uses these two types of data, presented by V. Rao, adds an additional branch to the segmentation network for image-level classification. The method also studies the weakly annotated images to learn to avoid features that are irrelevant for the segmentation task [95]. Deep Neural Network (DNN) is applied on the Pixel wise multimodal image representation that includes T1, T1c, T2, and Flair for the segmentation. DNN learns from each pixel of the image and segments the brain region more accurately [139]. Table 5 describes the overview of recent development for brain tumor segmentation.

State-of-the-art neuroimaging techniques are available for the detection of visible and invisible tumor cells. The variability in the shape and size of the tumor increases difficulties for automatic image segmentation. A hybrid Random Forest and Support Vector Machine (RF-SVM)-based method learns from the complex characteristics of the tumor lesion. RF-SVM consists of two-stage cascade in the first stage, random forest learns from the tumor label space and, at the second stage, the predicted features are fed into the SVM for classification. RF-SVM performs well as it is used solely for the segmentation [140].

Fully Convolutional Network (FCN) is used for segmentation of the tumor region and modifies the network with bounce structural chart to facilitate the semantic requirements for segmentation. Three-dimensional CNN is used for segmentation of the brain tumor. S. Kumar uses UNET and crops the image when fed into the network for better results [100]. The interactive deep-learning-based framework consists of the integration of CNNs into the bounding box and the scribble-based image segmentation pipeline is developed by G. Wang for tumor segmentation. The image-specific fine-tuning-based CNN’s model becomes more adaptive for specific test images [141]. 

The large size and dimensions of images (an image size up to gigabyte) and a limited amount of training data affect the performance of the Deep Convolutional Neural Network (DCNN). The convolutional neural network extracts the features and train their activation function through ImageNet knowledge, along with features selection, data augmentation, and feature pooling functions [76]. Convolutional Neural Network uses an encoder and decoder network with a singular hourglass structure for segmentation of the tumor region. Some preprocessing techniques are applied first and then the processed data is fed into the network. The hourglass method classifies the tumor into a core using one pass iteration [89]. Convolutional Neural Network has a powerful learning ability that learns attentive and contextual information when multiple deep layers of a variant structure are added to the network architecture, and produces more robust results for tumor segmentation. The risk of over-fitting for segmentation is reduced with the modified network and achieves a better Dice score for Brats 2018 data set [90]. Multi-Scale information requires brain image segmentation using boundary detection with the global context. The CNN uses down and upsampling of images to compute the features at a multi-scale level for semantic segmentation. The downsampling path requires a pooling operation which includes CNN, that is not desirable for segmentation tasks. The dense net is applied on a Brats 2017 dataset that excludes the pooling operation and adds delated convolutions, excluding the non-brain tissue for segmentation of the tumor region [91]. 2D fully convolutional network preforms better for segmentation with an increase in the depth of the architecture. Inception modules, convolutional layers, and the dense module were added in the U-Net architecture to the depth of the network and performance of the U-Net is computed. Deep U-Net architecture is trained on different image orientations without data augmentation techniques [92]. The 2D deep neural-network-based algorithm detects and segments the intra structure of tumors including enhancing, non-enhancing, necrosis and edema, forming multimodal MR brain images. Cascade U-net detects the tumor region and DCNN segments the patch base intra-tumor structure [93].

Fuzzy Logic with a Spiking Neuron Model (FL-SNM) is used for segmentation of the tumor region in MRIs. Modified Kuan Filter (MKF) is used to remove Poisson and Gaussian noise ftom the image before bringing it to the FL- SNM model. Random Search Algorithm (RSA) optimizes the image pixels and improves the Peak Signal-to-Noise Ratio (PSNR). Anisotropic Diffusion Filter (ADF) smooths the image and reduces the over-filtering problems. Afterwards, Fisher’s Linear Discriminant Analysis (FLDA) extracts the statistical texture features from the MRIs. The extracted features are transferred to the FL-SNM for the effective segmentation of the tumor region. Chicken Behavior-Based Swarm Intelligence (CSI) algorithm optimizes the weight value as weight and bias values are important in the FL-SNM model for tumor segmentation [94]. 

The segmentation of brain MRIs is implemented using the newly presented Fully Convolutional Residual Neural Network (FCR-NN), which is based on the linear identity of mappings. FCR-NN is a combination of optimizied residual and fully convolutional networks that efficiently segments low- and high-grade image features. In FCRe-NN, two different networks train the data, initially whole segmentation is performed and later on, tissue-based sub-region segmentation is achieved. FCR-NN enhances the overall Dice score for complete core and enhancing tumor [98]. 

Glioblastoma brain tumor segmentation is performed using convolutional neural networks with few layers and small receptive fields that minimizes the contextual and quality information for tumor segmentation. U-Net employs multiple layers for training and uses dynamic sampling of training data [99].

### 6.1. Feasibility Studies on Segmentation

Deep learning methods and models use a large amount of data for semantic segmentation of brain tumors, and it is a challenging task to acquire sufficient data for the training of models. The labeling of medical images requires domain knowledge expertise. Sharing the medical data of patients to a centralized location results in privacy, legal, data-ownership and technical challenges at the international level. The federated learning approach is used for semantic segmentation without sharing patient data by the multi-institutional collaboration. Federated learning provides better accuracy for semantic segmentation, with respect to the model that is trained on sharing data [145].

Tumor lesion location, use of Anti-Epileptic Drugs (AEDs) and the development of psychiatric symptoms have strong correlations among them. Treatment-Emergent Psychiatric Adverse Event (TE-PAEs) is possible through AED therapy and meets the conditions that includes onset within 4 weeks after AED therapy is perfromed, the absence of any other notorious possible concurrent cause, and disappearance upon drug discontinuation [146].

### 6.2. Proposed Approaches for Segmentation 

The diagnosis, planning, treatment, and evaluation of treatment outcome depends on accurate and reliable tumor segmentation. Fully Convolutional Neural Networks (FCNNs) and Conditional Random Fields (CRFs) are jointly used for the segmentation of tumor regions. Firstly, FCNNs-CRFs train FCNNs using slices and patches of 2D images. The parameters of FCNNs with image slices are used to train CRF as Recurrent Neural Networks (CRF-RNN), and image slices are used for the fine-tuning of FCNNs and CRF-RNN. 2D images patches are used to obtain coronal, axial and sagittal views, and voting-based fused-strategy is performed to combine these slices in tumor segmentation. The FCNNs-CRFs segment images into slice-by-slice orientation instead of patches which makes it much faster as compared to other existing segmentation models [1].

The variational model detects the saliency in MRIs and segments tumor regions. The variational model also detects the region of interest for the tumor. The proximal point algorithm solves the non-convex and non-smooth problems in the segmentation [147] to find a method for segmenting the brain tumor. The method consists of preprocessing, post-processing and a deep learning-based classification model. The model starts from preprocessing, which extracts the images patches for brain MRIs to achieve the gray level sequences of MRI patches that trains the deep learning network. The deep learning uses a stacked autoencoder to extract the high-level features of the image and uses the selected images patches for classification. Morphological filters are used for post-processing and convert the obtained result into a binary image for final segmentation result [113].

Multi-modal MRIs are used for brain tumor segmentation using automated generative models. The generative model is useful for healthy brain tumor tissues, the combination of spatial atlas-base for tissue prior and Gaussian mixture models for tissue modulation. To shape the core and complete tumors prior-to-tumor-based model, convolutional Restricted Boltzmann Machines (cRBMs) was presented by M. Agn [142]. The cRBMs model is effective for low and high-grade gliomas’ segmentation as it uses expert segmented images for training that do not use intensity information of images [142].

The Hybrid Pyramid U-Net (HPU-Net) model explores the contextual information of different region-based contexts. HPU-Net predicts pixel-level segmentation using global context information and produces good quality results for tumor segmentation. HPU-Net is based on multimodal tumor segmentation and performs end-to-end training and testing. The model uses downsampling and symmetrical upsampling paths and concatenates the features of up and downsampling at the symmetrical block. In the up-sampling process, multiple-scale features are extracted from each block and are added pixel-wise to recover the origin resolution. The integration of multi-scale, semantic and location information before the softmax layer increases the efficiency of tumor segmentation [142].

Brain tumor segmentation has received great attention in the domain of soft computing and medical images. Machine learning and deep learning methods require a large amount of data for their training that is expensive in the biomedical field. Different data augmentation techniques are available to expand the size of taring data to achieve better segmentation results. Generative Adversarial Networks (GANs)-based automatic data augmentation methods, presented by T. C. W. Mok and A. C. S. Chung, make the available annotated samples more efficient for deep-learning-methods [111]. The method consists of the coarse-to-fine generator that captures manifold training data and generates general augmented data for the segmentation [111]. Differential Evolution algorithm combined with OTUS is used to optimize the threshold value of the particular image and train the neural network for segmentation [112]. Deep learning technologies in the medical field improve the awareness of bio mechanisms for brain tumor segmentation. The segmentation of brain tumors is difficult due to variability in the size, shape, and location of tumor cells. The identification and segmentation of gliomas tumor from MRIs is a challenging task due to variabilities in tumor location, shape, spatial extent, intensity signature and the possible distance between normal and tumorized tissues. A novel, simple Fully Convolutional Network (FCN) segments the tumor efficiently and gives a faster runtime than other methods [106]. A Multiple Convolutional Neural Network-based framework with discrimination mechanisms was proposed by L. Zhao and K. Jia to overcome the segmentation problem, that includes accurate segmentation and protects the image form large and complex biases added to the MRIs. The 2D multiple CNNSs reduce the segmentation time for 3D voxel classification of brain tumors [108]. Another Multiscale Convolutional Neural Network that is based on statistical threshholding, segments the tumor region effectively. The statistical threshold method perfoms the coarse segmentation of the tumor. The multiscale convolutional neural network obtains the 2D multi-modality image that is roughly segmented by the statistical method for final tumor segmentation [102]. 

A generative adversarial network (voxel-GAN) addresses the data imbalance problems in the brain tumor’s segmentation as the majority of the voxels come from the healthy region and few voxels belong to the non-healthy or tumor region. 3D conditional Generative Adversarial Network (cGAN) consists of a segmentor and discriminatory segmentor to learn the segmentation labels from 3D MRIs and the discriminator differentiates the segmentor output in the ground truth data and the output that is artificially generated. The discriminator and segmentor networks are trained on newly generated weight adversarial loss to reduce the imbalance problem in the training data [104]. 3D Deep Convolutional Neural Networks (3D DNNs) are most popular for tumor segmentation as 3D DNNs have a strong learning capability with a large number of parameters for effective segmentation. 3D large kernel anisotropic network addresses problems that arise due to a large number of parameters, especially the selection of valid receptive fields which forms a large number of features that causes high computational cost and model overfitting. The 3D large kernel consists of an encoder and decoder, a large kernel encoder to make sure the valid receptive field is large enough and an anisotropic CNNs encoder is used to simulate the isotropic ones with fewer parameters [103]. Fully Convolutional Network (FCN) along with multi-task are presented by H. Shen for the automatic segmentation of brain tumor. Multi-task FCN extracts the contextual information at multi-levels using the symmetric-difference from multi-model MRIs. It integrates boundary information directly into the loss function and achieves efficient segmentation results [105].

Random Forest technique computes probabilities for multi-modality geometry, intensity and asymmetry feature sets for the supervised segmentation. Random Forest model also generates probability maps and these maps are used to refine the Markov random field for probabilistic segmentation. Advanced Normalization Tools (ANTs) and R Statistical (ANTsR) are used to investigate the learning capabilities of random forest for probabilistic segmentation [107].

### 6.3. Enhancement Approaches towards Segmentation 

The brain tumor develops due to the creation of abnormal cells in the brain tissue, and there are two types of brain tumors including benign and malignant tumors. The benign tumor does not affect human health but the malignant tumor has a lethal effect on the surrounding healthy and normal tissues in the brain that leads to the death of a patient. Early detection of tumor is necessary for treatment and patient survival. Segmentation of the tumor region is a challenging task due to the irregular shape and location of the tumor cell.

A kernel-based CNN combined with M-SVM presents an effective method for the enhancement and automatic segmentation of tumors. The method consists of preprocessing phase, features extraction method and tumor segmentation. Laplacian Of Gaussian (LOG) filtering method and Contrast Limited Adaptive Histogram Equalization are used for MRIs enhancement and extraction of features that are based on the shape, size and their location in the brain. The kernel-based CNN method uses MRIs and M-SVM to classify the tumor that is segmented by kernel-based CNN [109]. Stationary Wavelet Transform (SWT) and Growing Convolutional Neural Network are jointly used for a better segmentation of tumor region. SWT enhances the accuracy level of GCNN for segmentation [12].

A hybrid method, used for the segmentation of tumors by W. Deng, is a combination of a fully convolutional neural network and Dense Micro-block Difference Feature (DMDF) [110]. The Fisher vector encoding method analyzes the texture features to avoid rotational change and scale in texture images. The obtained local feature is fused to the Fully Convolutional Neural Network (FCNN) for fine boundary segmentation and then the de-convolutional skips the connection and a high-quality features map is obtained for segmentation [110].

### 6.4. Approaches toward Automatic Segmentation

The automatic segmentation of brain tumors into the whole tumor, core tumor and enhancing tumor form multi-model MRIs is dependent on tumor regions. The cascade of full CNNs decomposes the multi-class segmentation region into three binary segmentation regions. The cascade FCNNs work as the first segment for the whole tumor and bounding box of results is used for the segmentation of the core tumor. In the second stage, bounding box results of the core tumor are used to segment the enhancing tumor. The cascade of FCNNs consists of multiple layers of dilated and anisotropic convolutional filters and reduces the false-positive rate using multi-view fusion. The multi-scale prediction and residual connections of cascade FCNNs boost the segmentation performance [118].

Deep Learning (DL) and Multi-Atlas (MA) methods performed on Dual-Energy Computed Tomography (DECT) data have distinguished the healthy tissues from tumor tissues that are referred to as Organs-At-Risk (oARs). The Dual-Energy CT (DECT) dataset has high-resolution images as compared to single-energy CT. DL methods achieved better results for segmentation on DECT in comparison to single-energy CT for qualitative and quantitative analysis [148]. A 3D convolutional neural network deals with the partial volume averaging, inter-slice intensity variation and noise sensitivity. The intensity in homogeneity and intensity non-standardization is used to segment the tumor regions effectively. N3T-spline reduces the intensity and noise variation by correcting the bias field distortion and using a gray level co-occurrence matrix to extract the features from texture patches. 3D CNNs use these features and automatically segment the tumor into various abnormal tissues [121].

Structured Random Forest (SRF) and Bayesian Networks (BN)-based learning frameworks segment the multi-label images automatically. The structured random forest and Bayesian networks are embedded into multi-layer deep learning architecture and they cooperate for better learning of tumor features for multi-label segmentation. In the SRF-BN method, SRF performs pixel-level segmentation by exploring the contextual and structural information of the image, and BN supervises the statistical dependencies of image components at super pixel-level.

BN input probabilities maps are generated by SRF and original multi-model images are employed in each multi-layer of deep architecture. In the context of learning transfer from SRF to BN, BN performance has been improved gradually. In the next layer, the performance of SRF increases using original multimodal image and BN segmentation maps. In the SRF-BN method, both use the segmentation maps from the previous layer and the learning capabilities are increased in the networks. Thus better performance is achieved in the segmentation of tumors [97]. 

The U-Net base fully convolutional network measures the tumor’s level and automatically segments the tumor region into the whole, core and enhancing tumor [122]. 

The 2D Deep Convolutional Neural Networks (DNNs) automatically extracts the tumor into whole-tumor and intra-tumor regions’ in multimodal 3D MRIs. 2D convolutional neural network inspired by U-Net is modified using Generalized Dice Loss (GDL) and Weighted Cross-Entropy (WCE) as a loss function is used to address the class imbalance problems the tumor data. The proposed method was tested on BraTS 2018 dataset and had achieved a good dice score for Whole, Core and Enhancing tumor [114]. 

Deep Convolutional Neural Networks (DCNNs) use relatively small datasets for their training and data augmentation techniques are used to increase the performance of CNNs. The network structure of the CNNs is updated through flipping, scaling, image 3D rotation, adding noise at both training and testing times, and applying data augmentation techniques increase the performance of DCNNs in brain tumor segmentation [101]. 

Cascade’s fully convolution neural network is an effective method for image segmentation that splits multi-model MRIs into subhierarchy regions. 3D SE-inception network employs the 3D multi-model image data instead of 2D images. The 3D SE-inception uses dilated convolutional filters, and 3D Squeeze and Excitation structures for 3D segmentation. In the 3D SE-inception system, the bounding box results of whole tumor are used for the segmentation of the core tumor and bounding box results of core tumor are used for the segmentation of enhancing tumor [115].

The hybrid method of modified U-Net is combined with a domain-adapted version (DAU-Net) to segment the tumor by dividing the training samples in two domains. Firstly the preliminary tumor segmentation results are obtained and secondly, the domain invariant features are computed using modified U-Net [116].

A U-net neural network with three layers, one for the each region of interest, segments the tumor region into the whole, core and enhancing tumor effectively. The U-net model preprocesses the data of the patients before segmenting the tumor regions into the whole, core and enhancing tumor. The proposed multi-U-net model predicts the tumor location and survival time of the tumorized patient [117].

Convolutional neural network segments the tumor on the basis of multi-paths and is very effective for automatic segmentation as the multi-path CNNs is obtained using the contextual information in segmentation of multi-scale-regions of MR images. In the multi-path, CNNs spatial information is used to identify the healthy and tumorized regions of the brain [119]. 

Random Forest (RF) and Binary Decision Tree use multi-spectral MR images for efficient segmentation of the brain tumor region. RF-BDT preprocess the image dataset by reducing the effect of relative intensities and increase the features information at each voxel of the MR image [120].

Semi-Automatic Images Segmentation (SAMBAS) was presented by D. Gering for tumor segmentation in which Multi-Plane Reformat (MPR) is used to draw a long axis of the 3D segmented image. When 3D segmentation is performed on MPR, the 2D segmentation is updated in real-time. All necessary additional short axes, long axes, and other editing operations are drawn on the MPR plane. SAMBAS performs probability distribution in MPR segmentation and accelerates the process of 3D segmentation [123].

The deeply supervised neural network based on Holistically-Nested Edge Detection (HED) automatically segments the brain tumor from multi-model MRIs. The HED method works for binary edge detection of images for classification but also is applicable for multi-class tumor segmentation. The HED method segments the brain tumor into multiple classes that include whole, core and enhancing tumors [124].

## 7. Brain Tumor Evaluation

Positron Emission Tomography (PET) images tool is used for assessing brain tumors and differentiating tumor progression from reactive changes. The integration of Fluoro Ethhlyl Tyrosine and PET (FET-PET) method adds valuable information to MRIs for a better decision. Attenuation Correction term is used for acceptance of tumor in the FET-PET method. Deep-UTE and RESOLUTE methods generate CT-AC metrics more effectively. The Deep-UTE method produces more robust clinical metrics using CT-AC and overall patient survival time is increased. PET/MRIs’ attenuation correction in the Deep-UTE method is reliable for brain tumor evaluation due to better noise handling capability and less runtime properties [81]. 

## 8. Frameworks for Brain Tumor

**Figure 4:**
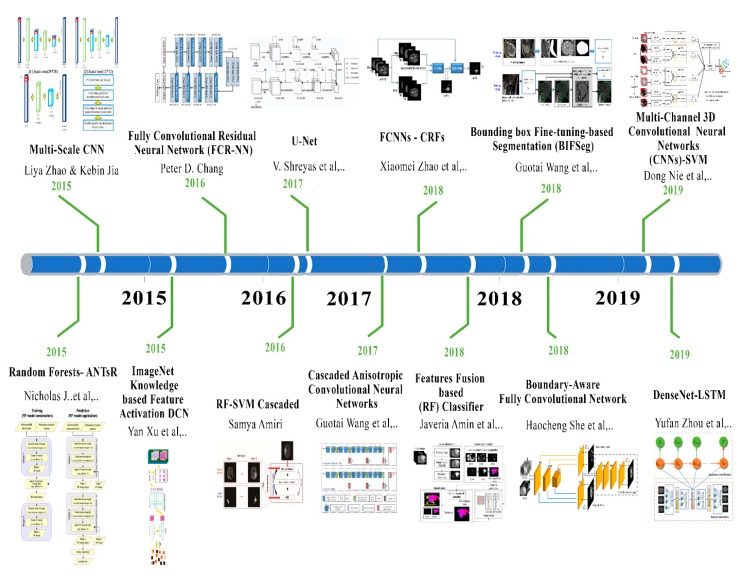
Deep learning development toward brain tumor through recent years.

The main aim of brain surgery is to perform the resectioning of tumors more accurately and preserve normal brain cells for the patient. The development of label-free and non-contact methods and frameworks is necessary to support the reliable resection of the tumor in real-time. Hyperspectral imaging is non-ionizing, label-free and non-contact. The deep-learning framework preprocesses the hyperspectral images in vivo brain tissues. The framework generates a thematic map that shows the parenchymal area of the brain and the location of the tumor is identified that helps the surgeon in successful and precise tumor resection [82]. Figure 4 shows the recent developments in deep learning for brain tumor analysis.

## 9. Discussion 

### 9.1. Overview 

Numerous papers were studied to conduct a review that shows how deep learning methods and techniques achieve state-of-the-art performance in every aspect of medical image analysis, especially in the field of brain tumor analysis, segmentation and classification. The large diversity of deep-learning-based architectures and methods is covered in this article. The pre-trained Convolutional Neural Network is used as a features extractor in various studies. The Capsule Network and Generative Adversarial Network (cGAN) has also been used for medical image analysis in various articles. These pre-trained networks download easily and can be directly applied to any format of medical images. Moreover, the existing approaches and systems use handcrafted features. In the last three years, for medical image analysis, an end-to-end trained CNNs approach has been preferred by researchers. It is reported that Convolutional Neural Networks (CNNs) have replaced traditional handcrafted machine learning methods and were integrated into existing medical image analysis pipelines. A large number of papers that are studied in this review, follow the above approach that is being practised in current standards.

### 9.2. Key Aspects of Successful Deep Learning Methods 

After reviewing the various papers, one would expect to be capable to distill the perfect deep learning architecture, approach, and method for individual tasks and application areas. The CNN-based architectures and methods would be top performers in most brain-tumor-based image analysis competitions. We can draw one striking conclusion that the exact architecture is not an important determinant for getting a good solution. We have observed, in different challenges including BraTS challenges (2015–2019), many researchers have used similar architectures in the same types of networks, but got extensively varying results [143,144]. Many researchers even added more layers in the CNNs network to increase the accuracy, which is the key aspect overlooked in expert knowledge. The researchers and groups that acquire good performance by applying deep learning methods and algorithms were able to do so using means outside the network such as the implementation of novel data augmentation and preprocessing techniques. In many BraTS challenges, researchers improved accuracy by adding normalization pre-processing steps that improve the generalization capabilities of the network without changing the CNN’s architecture. Different researchers focus on data augmentation techniques and strategies that make the CNN’s network more robust and they state that these strategies are very useful to obtain good performance. Data augmentation and pre-processing techniques are the key contributors to good solutions. Several researchers have observed that designing architectures for specific task properties attain better results than straightforward CNNs. Multi-view and multi-scale networks are examples of task-specific architectures that were encountered by the researchers several times. Network input size and receptive field are basic parts in designing a network (i.e., the input space area corresponds to a single output unit). The selected input size should fulfill the required context and resolution to solve the problem. The increment in the patch size to gain more context would not be beneficial without changing the receptive fields of the network. Another standard sanity check was performed by the researchers to assess the visual input of the network for the same task. If the researchers are domain experts and do not achieve good performance results then the need for modification in network architecture or input is high. The model hyper-parameter optimization (e.g., dropout rate, learning rate) aspect also affects the performance of the network. Disappointingly, there were no clear techniques or methods to assess the best set of hyper-parameters for empirical exercise. Researchers have also experimented Bayesian methods for hyper-parameters’ optimization but in the domian of brain image analysis, these methods have not been implemented till now.

### 9.3. Open Research Challenges, Limitations and Future Directions 

**Figure 5:**
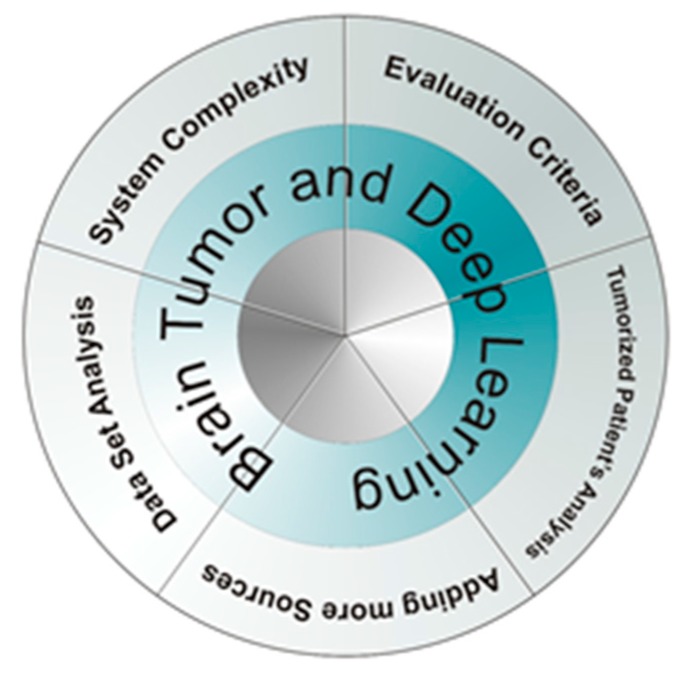
Open Research Challenges in brain tumor analysis.

The implementation of deep learning methods and algorithms in brain tumor image analysis presents numerous unique challenges. The lack of large training datasets is a challenging obstacle for deep learning methods. In the last decade, several PACS, MRIs and CT systems have been installed in various hospitals that generate tons of medical images. In some other fields, image data are used in well-structured digital archives that have a specific purpose. The PACS and CT systems are not broadly used in other fields of medicine such as pathology and ophthalmology. It has been observed that the number of available public datasets has increased gradually. Sophisticated text-mining techniques and methods are mandatory when writing reports on annotations or change structured labels in automated manners, where deep-learning-based methods and techniques are widely used. The introduction of structured labeling reports in the health domain, especially in brain tumor analysis, is expected to become easier in the future. It is predicted that, in future, the use of text-free and structured reports for training a network may increase rapidly, especially in the domain of brain tumor analysis. The researchers have asked domain experts (e.g., pathologists, radiologists) to make task-specific (e.g., segmentation, prediction, classification) and text-free reports from image data to train deep learning algorithms. The labeling of tumorized images is not only time-consuming but it also requires a high level of expertise that is challenging in brain tumor analysis.The training of systems based on deep learning algorithms, performing the segmentation of tumors, mostly in 3D networks, needs slice-by-slice annotations that are a not only challenging but also time-consuming task. The effeicient learning of deep learning methods from a limited amount of image data is also a major limitation of deep learning algorithms. Various researchers have trained their 3D segmentation models using only 2D segmentation [149]. To evaluate tumor analysis algorithms and to predict a tumor in brain MRIs, BraTS datasets are widely used. In this dataset, four types of tumor are annotated by radiologists. Training a deep learning system using these data needs additional consideration for modeling uncertainty and noise in the standard reference. A few researchers have provided solutions by incorporating label uncertainty directly in the loss function, but this is still an open challenge. Another problem related to data is class-imbalance. For example, data augmentation techniques are used to generate new lesions of brain tumors through scaling and rotation but this may cause class-imbalance. Pereira evaluates the data augmentation strategies for tumor lesion segmentation to combat class imbalance [150]. However, most deep learning methods and architecture in brain tumor analysis still deal with patch classification, where the anatomical location of the patch remains unknown for the network. A possible solution for this is that the entire image is fed into the deep network and using various methods, the learning process of network is achieved, for example, Milletari et al., designed a loss function that is based on the Dice coefficient [151]. However, if the network has a small receptive field for the entire image data, then there is no advantage for deep networks. The feeding of a full image into the network is not feasible sometimes due to a few constraints such as limited memory, GPU, and bandwidth, as the size of brain tumor images is generally in the gigapixels range. Another research challenge is that, generally, researchers have employed the same fixed size for a kernel to perform image slicing, which may hide some useful information from another region that is ignored by the kernel. A few researchers have used a variable size of kernel to slice the image data but more work is needed in this area. Figure 5 describes the open research challenges in brain tumor analysis.

## Figures and Tables

**Table 1:** Data sources and their acquisition methods.

Sr. No	Paper	Acquisition Method	Dataset Sources
1.	Xiaomei Zhao et al. [1].	Online repository	BraTS 2013, BraTS 2015 and BraTS 2016
2.	Mamta Mittal et al. [12].	Online repository	BRAINIX medical images. (https://www.medicalimages.com/search/brain.html)
3.	Guotai Wang et al. [26].	Online repository	BraTS 2018
4.	Mikael Agn1 et al. [30].	Online repository	BraTS (http://braintumorsegmentation.org/)
5.	M. Zhou et al. [37].	Not given	Not Mentioned
6.	Subhashis Banerjee et al. [67].	Online repository	TCGA-GBM, TCGA-LGG (https://wiki.cancerimagingarchive.net/display/Public/TCGA-LGG)
7.	Yufan Zhou et al. [68].	Custom-developed	Proprietary Dataset. The public dataset [5] includes 3064 (2D) slices of brain MRI from 233 patients.
8.	Nyoman Abiwinanda et al. [69].	Online repository	Ffigshare Cheng (Brain Tumor Dataset, 2017)
9.	Esther Alberts et al. [70].	Online repository	The Cancer Imaging Archive” (TCIA) (https://www.cancerimagingarchive.net/)
10.	Ali ARI [71].	Not given	Not Mentioned
11.	Sajid Iqbal1 et al. [72].	Not given	Not Mentioned
12.	Yota Ishikawa et al. [73].	Not given	Not Mentioned
13.	Heba Mohsen et al. [74].	Custom developed	Harvard Medical School website (http://med.harvard.edu/AANLIB/)
14.	Justin S. Paula et al. [75].	Custom-developed	Publically available Nanfang Hospital, Guangzhou, China, and General Hospital, Tianjing Medical University
15.	Yan Xu et al. [76].	Online repository	TCGA (https://wiki.cancerimagingarchive.net/display/Public/TCGA-LGG)
16.	Kaoutar B. Ahmed et al. [77].	Online repository	H. Lee Moffitt Cancer Research Center
17.	A. R. Deepa1 & W. R. Sam Emmanuel [78].	Online repository	BraTS 2015
18.	Mustafa Rashid Ismael [79]	Online repository	BraTS
19.	Renhao Liua et al. [80].	Custom d developed	H. Lee Moffitt Cancer Research Center
20.	Nøhr Ladefoged et al. [81].	Custom-developed	PET/MRI system (Siemens Biograph mMR, Siemens Healthcare, Erlangen, Germany) (Delso et al., 2011) between February 2015 and October 2017, and 86 FET PET
21.	Himar Fabelo et al. [82].	Custom-developed	The intraoperative hyperspectral (HS) acquisition system was employed to create the HS image database.
22.	Yannick Suter1 et al. [83].	Online repository	BraTS 2018
23.	Yuexiang Li and Linlin She [84].	Online repository	BraTS 17
24.	Dong Nie et al. [85].	Custom-developed	Glioma image database (collected during 2010–2015) of Huashan hospital, Shanghai, China
25.	Javeria Amin1 et al. [86].	Online repository	BraTS 2012
26.	Lina Chato and Shahram Latifi [87]	Online repository	BraTS 2017
27.	Virupakshappa & Basavaraj Amarapur [88]	Not given	Not Mentioned
28.	Eze Benson et al. [89].	Online repository	BraTS 2018
29.	Chenhong Zhou et al. [90].	Online repository	BraTS 2018
30.	Richard McKinley et al. [91].	Online repository	2017 BraTS
31.	Geena Kim [92].	Online repository	BraTS2017
32.	Yan Hu and Yong Xia [93]	Online repository	BraTS 2017
33.	Aparna Natarajan& Sathiyasekar Kumarasamy [94]	Not given	Not Mentioned
34.	Pawel Mlynarskia et al. [95].	Online repository	BraTS 2018
35.	Parnian Afshar et al. [96].	Not given	Not Mentioned
36.	Samya AMIRI [97]	Online repository	BraTS
37.	Peter D. Chang [98]	Online repository	2016 BraTS
38.	Fabian Isensee et al. [99].	Custom-developed	Not Mentioned
39.	Sanjay Kumar et al. [100].	Online repository	BraTS Dec 2017
40.	Guotai Wang et al. [101].	Not given	Not Mentioned
541	Yun Jiang et al. [102].	Online repository	BraTS2015
42.	Dongnan Liu et al. [103].	Online repository	BraTS17
43.	Mina Rezaei et al. [104].	Online repository	BraTS-2018 ISLES-2018 (http://www.isles-challenge.org/)
44.	Haocheng Shen et al. [105].	Online repository	BraTS15, BraTS13
45.	V. Shreyas and Vinod Pankajakshan [106]	Online repository	BraTS
46.	Nicholas J et al. [107].	Online repository	MICCAI 2013 BraTS
47.	Liya Zhao and Kebin Jia [108]	Online repository	BraTS
48.	R. Thillaikkarasi & S. Saravanan [109]	Not given	Not Mentioned
49.	Wu Deng1 et al. [110].	Online repository	BraTS 2015
50.			
51.	Tony C. W. Mok et al. [111].	Online repository	BraTS15
52.	Anshika Sharma et al. [112].	Online repository	IBSR data set Cyprus (http://www.medinfo.cs.ucy.ac.cy/)
53.	Zhe Xiao et al. [113].	Custom-developed	MRIs from real patients in West China Hospital
54.	Adel Kermi et al. [114].	Online repository	BraTS’2018
55.	Hongdou et al. [115].	Online repository	BraTs 2018
56.	Lutao Dai1 et al. [116].	Online repository	BraTS 2018
57.	Eric Carver et al. [117].	Online repository	BraTS
58.	Guotai Wanget al. [118].	Online repository	BraTS 2017
59.	Sara Sedlar [119]	Online repository	BraTS 2017
60.	Zoltan Kap et al. [120].	Online repository	BraTS 2016
61.	G. Anand Kumar and P. V. Sridevi [121].	Online repository	BraTS 2015
62.	Hao Dong et al. [122].	Online repository	BraTS 2015
63.	David Gering et al. [123].	Online repository	2018 BraTS
64.	Reza Pourreza et al. [124].	Online repository	BraTS 2017
65.	Caulo et al. [125].	Custom developed Jan 2008–Sep 2012	University G. d’Annunzio of Chieti-Pescara, Chieti, Italy
66.	Cheng et al. [126].	Custom-developed 2005–2010	Nanfang Hospital and General Hospital, Tianjin Medical University
67.	Wang et al. [127].	Custom-developed May 2004–Nov 2011	Hospital of Xi’an Jiaotong University
68.	Chaddad [128].	Online repository	Cancer Imaging Archive (http://www.cancerimagingarchive.net/)
69.	Rajini et al. [129].	Custom-developed	Department of Radiology, Rajah Muthiah Medical College Hospital (RMMCH), Tamil Nadu, India
70.	Javed et al. [130].	Online repository	brain database http://www.med.harvard.edu/AANLIB/home.html
71.	Al-Shaikhli et al. [131].	Online repository	Brain web for simulated brain database (http://brainweb.bic.mni.mcgill.ca/brainweb/)
72	Lahmiri et al. [132].	Online repository	Harvard Medical School (http://www.med.harvard.edu/aanlib/home.html)
73	Lin et al. [133].	Custom-developed Jan 2006–Dec 2012	National Defense Medical Center, Taipei, Taiwan, Republic of China
74	Xiangmao Kong et al. [134].	Online repository	BraTS 2015 and BraTS 2017

**Table 2:** Overview of papers using deep learning for brain tumor classification.

Study	Method	Proposed Solution and Preprocessing Approach	Software’s/Tools/Languages/ Libraries used for Simulation and Implementation	Evaluation
Subhashis Banerjee et al. [67].	Deep Convolutional Neural Networks (ConvNets) using multi-sequence MR images.		Terser flow and Python	Accuracy = 97%
Yufan Zhou et al. [68].	Convolutional Neural Networks	DenseNet-RNN, DenseNet-LSTM, DenseNet-DenseNET	Tensor Flow, Nvidia Titan Xp GPU	Accuracy = 92.13%
Nyoman Abiwinanda et al. [69].	Convolutional Neural Network	AlexNet,VGG16,ResNet	Matlab	Accuracy = 84.19%
Esther Alberts et al. [70].	SVM, RF, KNN, LOG, MLP and PCA	LBP, BRIEF and HOG	Not Mention	Accuracy = 83%
Ali ARI & Davut HANBAY [71]	Convolutional Neural Network	ELM-LRF	MATLAB 2015	Accuracy = 97.18%
Yota Ishikawaet et al. [73].	Deep Convolutional Neural Networks	BING objectness estimation, Voronoi diagram, Binarization, Watershed transform	Not Mention	Accuracy = 98.5%
Heba Mohsen et al. [74].	Deep Neural Network	Discrete Wavelet Transform (DWT), Principal Components Analysis (PCA)	MATLAB R2015a and Weka 3.9	Accuracy = 96.97%
Justin S. Paula et al. [75].	Convolutional Neural Network, Fully Connected Neural Network, Random Forests		Not Mention	Accuracy = 91.43%
Yan Xu et al. [76].	Deep Convolutional Activation Features	Deep Convolutional Activation Features trained by ImageNet knowledge	Not Mention	Accuracy = 97.5%
Parnian Afshar et al. [96].	Convolutional Neural Networks(CNNs)	Capsule Networks (CapsNets)	Python 2.7 and Keras library	Accuracy = 86.56%

**Table 3:** Overview of papers using deep learning for brain tumor Prediction.

Study	Method	Proposed Solution and Preprocessing Approach	Software’s/Tools/Languages/ Libraries used for Simulation and Implementation	Evaluation
Ali ARI & Davut HANBAYaks [71].	Convolutional Neural Network	ELM-LRF	MATLAB 2015	Accuracy = 97.18%
Yannick Suteret al. [83].	3D-convolutional neural networks (CNNs)	Support Vector Classifier (SVC), Hand-Crafted Features	Scikit-learn3 version 0.19.1.	Accuracy = 72.2%
Yuexiang Li & Linlin Shen [84].	CNN	Multi-view Deep Learning Framework (MvNet) and SPNet	PyTorch Toolbox	Accuracy =88.00%
Dong Nie et al. [85].	3D convolutional neural networks (CNNs)	Multi-Channel Architecture of 3D convolutional neural networks and SVM	Not Mention	Accuracy = 90.66%
Javeria Aminrt et al. [86].	Random forest (RF) classifier	Gabor Wavelet Features (GWF), Histograms of Oriented Gradient (HOG), Local Binary Pattern (LBP) and segmentation based Fractal Texture Analysis (SFTA) features	DWI and FLAIR	Dice Scores Complete = 0.91 Non-Enhancing = 0.89 Enhancing = 0.90
Lina Chato & Shahram Latifi [87].	Convolutional Neural Network (CNN), Linear Discriminant	Support Vector Machine (SVM), K-Nearest Neighbors (KNN), Linear Discriminant, Tree, Ensemble and Logistic Regression	Not Mention	Accuracy = 68.8%
Virupakshappa & Basavaraj Amarapur [88].	Adaptive Artificial Neural Network (AANN)	Modified Level Set approach	MATLAB	Accuracy = 98%

**Table 4:** Overview of papers using deep learning for brain tumor Deep Features, Evaluation and Framework.

Area	Study	Method	Proposed Solution and Preprocessing Approach	Software’s/Tools/Languages/ Libraries used for Simulation and Implementation	Evaluation
**Deep Features**	Kaoutar B. Ahmed et al. [77].	Convolutional Neural Networks (CNNs)	Fine-Tuning	Weka	Accuracy = 81%
	A. R. Deepa & W. R. Sam Emmanuel [78].		Fused Feature Adaptive	MATLAB	Accuracy = 99.84
	Mustafa Rashid Ismael [79].	deep neural networks	Stacked Sparse Autoencoder (SSA) and Softmax	Not Mention	Accuracy = 94%
	Renhao Liua et al. [80].	Deep Convolutional Neural Networks	Pre-trained CNN as a feature extractor to get deep feature representations for brain tumor magnetic resonance images.	Weka	Accuracy = 95.4%
**Evaluation**	Nøhr Ladefoged et al. [81].		RESOLUTE and DeepUTE		Precision = 0.67
**Frameworks**	Himar Fabelo et al. [82].	2D convolutional neural network		TensorFlow and Titan-XP NVIDIA GPU	Accuracy = 80%

**Table 5:** Overview of papers using deep learning for brain tumor segmentation.

Study	Method	Proposed Solution and Preprocessing Approach	Softwares/Tools/Languages/ Libraries used for Simulation and Implementation	Evaluation
Xiaomei Zhao et al. [1].	Fully Convolutional Neural Networks (FCNNs)	Integration of FCNNs and CRFs	Tesla K80 GPUs and Intel E5-2620 CPUs	Dice Scores Complete = 0.84 Core Tumor = 0.67 Enhancing = 0.62
Mamta Mittal et al. [12].		Stationary Wavelet Transform (SWT) and the new Growing Convolution Neural Network (GCNN).	Not Mention	Accuracy = 98.6 Precision = 0.9881 Recall = 0.9823
Yan Xu et al. [76].	Deep Convolutional Activation Features(CNNs)	CNN Activations Trained by ImageNet to Extract Features through Feature Selection, Feature Pooling, and Data Augmentation	Not Mention	Accuracy = 84%
Eze Benson et al. [89].	Convolutional Neural Network (CNN)	Singular Hourglass Structure	NVIDIA TITAN X GPU	Coefficient = 92%
Chenhong Zhou et al. [90].	Convolutional Neural Network	OM-Net MC-baseline and OM-Net from multiple aspects to further promote the performance.	Not Mention	Dice Scores Enhancing = 0.8136 Whole Tumor = 0.909 Core Tumor = 0.8651
Geena Kim [92].	2D Fully Convolutional Neural Networks	double convolution layers, inception modules, and dense modules were added to a U-Net to achieve a deep architecture	Not Mention	Dice Scores Enhancing = 0.75 Whole Tumor = 0.88 Core Tumor = 0.73
Yan Hu & Yong Xia [93].	Deep Convolutional Neural Network	3D Deep Neural Network-based Algorithm Cascaded U-Net	NVIDIA GTX 1080	Dice Scores Enhancing = 0..55 Whole Tumor = 0.81 Core Tumor = 0.69
Aparna Natarajan & Sathiyasekar Kumarasamy [94].	Fuzzy Logic with Spiking Neuron Model (FL-SNM)		MATLABR2017	Accuracy = 94.87%
Peter D. Chang [98].	Fully Convolutional Neural Networks	Fully Convolutional Residual Neural Network (FCR-NN)	MATLAB R2016a	Dice Scores Complete = 0.87 Core Tumor = 0.81 Enhancing = 0.72
Fabian Isensee et al. [99].	Convolutional Neural Networks	UNet Architecture	Pascal Titan X GPU	Dice Scores Whole = 90.1 Core Tumor = 90.0 Enhancing = 84.5
Sanjay Kumar et al. [100].	Fully Convolution Neural Networks	UNET Architecture	Not Mention	Accuracy = 89%
Guotai Wang et al. [101].	Convolutional neural networks (CNNs)	Fine-tuning-based Segmentation (BIFSeg)	NVIDIA GPU	Accuracy = 88.11%
Yun Jiang et al. [102].	Convolutional Neural Networks	Statistical Thresholding and Multiscale Convolutional Neural Networks (MSCNN)	Not Mention	Dice Coefficient = 86.6% Predictive Positivity Value (PPV) = 88.6% Sensitivity Coefficient = 85.2%
Dongnan Liu et al. [103].	Deep Convolutional Neural Network (DNN)	3D Large Kernel Anisotropic Network	CBICA’s Image Processing Portal	Dice Scores Whole = 0.86 Core Tumor = 0.81 Enhancing = 0.793
Mina Rezaei et al. [104].	3D Conditional Generative Adversarial Network (cGAN)	Adversarial Network, named Voxel-GAN	Keras library and Tensorflow	Dice Scores Whole = 0.84 Core Tumor = 0.79 Enhancing = 0.63 Dice = 0.83 Hausdorff = 9.3 Precision = 0.81 Recall = 0.78
Haocheng Shen et al. [105].	Fully Convolutional Network (FCN)	Boundary-Aware Fully Convolutional Network	Keras and Theano	Dice Scores Complete = 88.7 Core Tumor = 71.8 Enhancing = 72.5
V. Shreyas and Vinod Pankajakshan [106].	Simple Fully Convolutional Network (FCN)	U-Net	Uadro K4000 GPU	Dice Scores Whole = 0.83 Core Tumor = 0.75 Enhancing = 0.72
Nicholas J et al. [107].	Random Forests	Random Forests with ANTsR	ANTsR Package, CMake Tool, R-code	Dice Scores Complete = 0.87 Core Tumor = 0.78 Enhancing = 0.74
Liya Zhao & Kebin Jia [108].	Convolutional Neural Networks (CNNs)	Multi-Scale CNN Architecture of tumor Recognitionon 2D slice and Multiple Intermediate Layers in CNNs	Not Mention	Dice Accuracy = 0.88%
R. Thillaikkarasi & S. Saravanan [109].	CNN with M-SVM	Novel Deep Learning Algorithm (Kernel-based CNN) with M-SVM	Not Mention	Accuracy = 84%
Wu Deng et al. [110].	Convolutional Neural Network	Dense Micro-block Difference Feature (DMDF) and Fisher vector Encoding Non-quantifiable local feature FCNN and Fine Feature Fusion Model	GPU NVIDIA GeForce GTX1070, Ubuntu 16.04 LST 64-Bit operating System	Accuracy = 90.98%
Tony C. W. Mok et al. [111].	Generative Adversarial Networks	Novel automatic data augmentation Coarse-to-Fine Generator to capture the Manifold, Coarse-to-Fine Boundary-Aware Generator CB-GANs	Nvidia GTX1080 Ti GPU	Dice Scores Complete = 0.84 Core Tumor = 0.63 Enhancing = 0.57
Anshika Sharma et al. [112].	Neural Network	Differential Evolution algorithm Embedded with OTSU method Hybridization of Differential Evolution(DE) and OTSU	MATLABR2012a	Accuracy = 94.73%
Zhe Xiao et al. [113].		Coarse-to-Fine and ’Stacked Auto-Encoder’ (SAE). Stacked Denoising Auto Encoder SDAE	Not Mention	Accuracy = 98.04%
Adel Kermi et al. [114].	2D Deep Convolutional Neural Networks (DNNs)	Weighted Cross-Entropy (WCE) and Generalized Dice Loss (GDL) U-net	intel Xeon E5-2650 CPU@ 2.00 GHz (64 GB) and NVIDIA Quadro 4000–448 Core CUDA (2 GB) GPU.	Dice Scores Whole = 0.86 Core Tumor = 0.80 Enhancing = 0.78
Hongdou Yao et al. [115].		Cascaded FCN	GTX 1080Ti GPU	Dice Scores Whole = 0.86 Core Tumor = 0.73 Enhancing = 0.63
Lutao Dai et al. [116].	Deep Convolution Neural Networks	Integration of modified U-Net and its domain-adapted version (DAU-Net).	XGBoost	Dice Scores Whole = 0.91 Core Tumor = 0.85 Enhancing = 0.80
Eric Carver et al. [117].	U-net Neural Network		XGBboost	Dice Scores Whole = 0.88 Core Tumor = 0.76 Enhancing = 0.71
Guotai Wang et al. [118].	Convolutional Neural Networks	Cascade Fully Convolutional Neural Network with multiple layers of Anisotropic and dilated Convolution Filters	NVIDIA TITAN X GPU	Dice Scores Whole = 0.83 Core Tumor = 0.90 Enhancing = 0.78
Sara Sedlar [119].	Convolutional Neural Network (CNN	Multi-Path Convolutional Neural Network (CNN)	nVidia’s GeForce GTX 980 Ti (6 GB) GPU and Intel Core i7-6700K CPU @ 4.00 GHz (32 GB).	Dice Scores Whole = 0.84 Core Tumor = 0.69 Enhancing = 0.60
Zoltan Kap et al. [120].		Decision Trees and Random Forest technique	Not Mention	Dice score = 80.1% Sensitivity = 83.1% Specificity = 98.6%
G. Anand Kumar & P. V. Sridevi [121].	3D Convolutional Neural Network (3DCNN)	EGLCM Feature Extraction to Assess, Evaluate and Produce accurate predictions and detailed segmentation maps.	MATLABR2017a	Not Mention
Hao Dong et al. [122].	Fully Convolutional Networks	U-Net based Deep Convolutional Networks	NVIDIA Titan X (Pascal)	Dice Scores Complete = 0.86 Core Tumor = 0.86 Enhancing = 0.65
David Gering et al. [123].	Convolution Neural Network	Multi-Plane Reformat (MPR)	TensorFlow and Neural Networking API Keras	Dice Scores Active= 0.76 Core Tumor = 0.86 Whole = 0.89
Reza Pourreza et al. [124].	Deeply-Supervised Neural Network	Holistically-Nested Edge Detection (HED) Network	Caffe library Python and NVIDIA Titan Xp graphic card	Dice Scores Whole = 0.86 Core Tumor = 0.60 Enhancing = 0.69
Samya AMIRI [140].		Random forest (RF) based Learning Transfer to SVM RF-SVM cascaded	MATLAB	Mean Dice index Secore = 72.0%
Guotai Wang et al. [141].	Deep Convolutional Neural Networks (CNNs)	3D Unet, Cascaded Network of WNet, TNet and ENet	NVIDIA TITAN X GPU	Dice Scores Whole = 90.21 Core Tumor = 85.83 Enhancing = 79.72
Mikael Agn et al. [142].	Gaussian Mixture Model Combined with a Spatial Atlas-based Tissue Prior Generative Model	Convolutional Restricted Boltzmann Machines (cRBMs)	MATLAB 2014b.	Dice Scores Complete = 87 Core Tumor = 82 Enhancing = 70
Xiangmao Kong et al. [134].	U-Net	Novel Hybrid Pyramid U-Net (HPU-Net) Model for Pixel-Level Prediction	NVIDIA Titan X GPU	Dice Scores Complete = 0.90 Core Tumor = 0.71 Enhancing = 0.78 Predictive Positivity Value (PPV) Complete = 0.91 Core Tumor = 0.87 Enhancing = 0.93 Sensitivity Complete = 0.96 Core Tumor = 0.79 Enhancing = 0.67
Richard McKinley et al. [143].	Convolutional Neural Network (CNN)	Densenet and DeepSCAN	Not Mention	Dice Scores
Pawel Mlynarskia et al. [144].		Deep Learning Fully-Annotated and Weakly-Annotated	TensorFlow	Accuracy = 85.67%
